# P80 No immunologist, no problem: penicillin allergy de-labelling pilot in a London district general hospital

**DOI:** 10.1093/jacamr/dlaf230.087

**Published:** 2025-12-04

**Authors:** Tamara Hamoodi, Douglas Izzard, Jashmin Patel, Sonia Jeyaseelan, Imran Qureshi

**Affiliations:** Croydon Health Services, Croydon, UK; Croydon Health Services, Croydon, UK; Croydon Health Services, Croydon, UK; Croydon Health Services, Croydon, UK; Croydon Health Services, Croydon, UK

## Abstract

**Background:**

About 10% of the general population believes they have a penicillin allergy, but evidence shows that over 90% of those labelled do not truly have the allergy. Mislabelled penicillin allergies drive unnecessary use of broad-spectrum antibiotics (BSAs) and increased risks of antimicrobial resistance, *Clostridioides difficile* and increased healthcare costs. To mitigate these risks, the antimicrobial stewardship (AMS) team at Croydon University Hospital introduced a de-labelling protocol- the first of its kind in Southwest London, without an onsite immunologist. This initiative aims to accurately identify true allergies through systematic assessment.

**Objectives:**

to identify patients with low-risk penicillin allergy labels (PALs) for de-labelling from history alone or by offering a drug provocation test (DPT), restore appropriate first-line penicillin use and reduce reliance on broad-spectrum alternatives. Training on accurate allergy documentation and appropriate prescribing was included.

**Methods:**

A prospective observational study was conducted by the AMS team on the acute medicine ward over four weeks (July- August 2025). Patients with PALs were identified from the pharmacy care organizer of Cerner and risk-stratified in line with BSACI recommendations. Low-risk allergies (non-allergic symptoms, tolerated penicillin since documentation, or family history) were de-labelled on history or offered a DPT. Patients with severe asthma, COPD, heart failure, angioedema after penicillin, or those requiring hospital treatment were deemed as high risk and allergy status was refined or retained. Outcomes were documented for analysis, including final allergy status, DPT eligibility/exclusions and communication to primary care.

**Results:**

In total, 28 PALs were reviewed. 46% (*n*=13) were removed based on clinical history alone. A further 7% (*n*=2) could have been removed but patients refused. 25% (*n*=7) were refined, for example to include specific sensitivity to certain penicillins or the appropriateness of cephalosporins. Twenty-five percent (*n*=7) had low risk allergies eligible for a DPT. Of these, 14% (n=4) met the exclusion criteria due to a NEWS greater than 1 and a further 11% (*n*=3) refused consent. Fifty percent of PALs reviewed required communication to the GP to amend the allergy. Only 11% (*n*=3) had the update documented in their discharge summary. Among patients successfully de-labelled, penicillin prescribing was reinstated during admission where clinically indicated, avoiding unnecessary broad-spectrum alternatives.

**Conclusions:**

A structured PADL approach in a district general hospital safely removed or refined many PALs with most changes achieved through history alone. De-labelling enabled a reduction in BSA use by restoring safe first-line penicillin options. Barriers included restrictive eligibility criteria, patient refusal and unreliable communication to primary care. Optimizing selection criteria, embedding PADL into routine AMS activity and strengthening GP communication pathways are essential to scale this intervention and maximize stewardship benefits.

